# The plant defensin gene *AtPDF2.1* mediates ammonium metabolism by regulating glutamine synthetase activity in *Arabidopsis thaliana*

**DOI:** 10.1186/s12870-019-2183-2

**Published:** 2019-12-16

**Authors:** Junyue Yao, Jin-Song Luo, Yan Xiao, Zhenhua Zhang

**Affiliations:** 1 Southern Regional Collaborative Innovation Center for Grain and Oil Crops in China, College of Resources and Environmental Sciences, Hunan Agricultural University, Changsha, China; 2 Hunan Provincial Key Laboratory of Farmland Pollution Control and Agricultural Resources Use, Hunan Provincial Key Laboratory of Nutrition in Common University, National Engineering Laboratory on Soil and Fertilizer Resources Efficient Utilization, Changsha, 410128 China

**Keywords:** Ammonium metabolism, *Arabidopsis thaliana*, GLN1.3, GLN1.5, PDF2.1, Plant defensins

## Abstract

**Background:**

In plants, ammonium metabolism is particularly important for converting absorbed nitrogen into amino acids. However, the molecular mechanism underlying this conversion remains largely unknown.

**Results:**

Using wild type *Arabidopsis thaliana* (Col-0) and *AtPDF2.1* mutants (*pdf2.1–1* and *pdf2.1–2*), we found that the small cysteine-rich peptide AtPDF2.1, a plant defensin, is involved in regulating ammonium metabolism in the shoot. Ammonium significantly induced the expression of *AtPDF2.1* in the shoot and root, particularly in root xylem vascular bundles, as demonstrated by histochemical analysis. Subcellular localization analysis revealed that AtPDF2.1 was localized to the cell wall. Ammonium concentration was higher in the shoot of mutants than in the shoot of Col-0, but no differences were found for total nitrogen content, root ammonium concentration, and the expression of the ammonium transporter gene *AtAMT2.1*. The activity of glutamine synthetase was significantly decreased in mutants, and the glutamine synthetase family genes *GLN1.3* and *GLN1.5* were significantly downregulated in mutants compared to Col-0. The activity of nitrate reductase showed no difference between mutants and Col-0.

**Conclusions:**

Overall, these data suggest that *AtPDF2.1* affects ammonium metabolism by regulating the expression of *GLN1.3* and *GLN1.5* through a yet unidentified mechanism.

## Background

Plant defensins (PDFs) are small cysteine-rich peptides, usually composed of an N-terminal signal peptide, C-terminal variable region, and cysteine-rich domain [1, 2]. Nuclear magnetic resonance analysis of the structure and homology of RsAFP1 in radish revealed that this defensin had a common cysteine-stabilized alpha beta conformation with four disulfide bonds on an alpha helix in reverse parallel with three beta angles [3]. Plant defensins are ubiquitous in plants and animals and mediate innate nonspecific immune responses [4]. Most of the defensins identified in plants have broad-spectrum antimicrobial activities, inhibiting amylase and blocking ion channels [2, 5]. Two families of PDFs have been reported in *Arabidopsis thaliana*. The first family contains seven members with high homology (PDF1.1, PDF1.2a, PDF1.2b, PDF1.2c, PDF1.3, PDF1.4, and PDF1.5), five of which are very similar (PDFs 1.1 to 1.3). In addition, the predicted mature structures of PDF1.2a, b, and c are similar. The members of the second family (PDFs 2.1 to 2.6) are also very close. While PDFs 2.1, 2.3, and 2.6 appeared in a tandem array, PDF 2.2 and the other genes were not in the same branch [2]. Previous studies on different PDF genes (*PDF1.1*, *1.2*, *2.1*, *2.2*, and *2.3*) have shown their organ-specific expression patterns [2, 6]. Recent studies have evidenced that PDFs are also involved in abiotic stress response, as their expression levels are induced by cold, drought, and heavy metal stresses [7–9], and PDF2.3 is likely related to potassium ion homeostasis [10]. Latest research revealed that PDFs mediate cadmium tolerance and accumulation in rice and *A. thaliana* [11, 12].

Small peptides can be used as signal molecules to regulate nitrogen (N) response and stress adaptation [13, 14]. Small C-terminal-encoded peptides (CEPs) [15], for instance, are produced by N-starved roots and translocated to the shoot where they interact with leucine-rich repeat receptor kinase CEP receptor 1/2 (CEPR1/2) [13]. Rhizobium-induced xylem mobile CLAVATA3/EMBRYO SURROUNDING REGION-RELATED (CLE) peptides have been shown to inhibit nodulation in legumes [16]. Although it is not clear whether PDFs interact with N in *A. thaliana*, we hypothesize that small PDFs may also function as signal molecules in regulating N metabolism.

N is an essential mineral element for plants and plays an important role in plant growth and development. It is not only a component of nucleic acids, amino acids, and proteins; it also participates in carbon assimilation during photosynthesis as a component of chlorophyll, and an interaction between N and phosphorus has been reported in rice [17]. Nitrate and ammonium are the main forms of N uptake by plants. When nitrate is absorbed by plants, a part of it is directly transported to the aerial parts or stored in vacuoles in the root cells, and another part is converted to ammonium or integrated into amino acids for metabolism or transportation to the aerial parts [18].

In agricultural production, the application of N fertilizer generally has a substantial yield-increasing effect [19, 20]. However, the low utilization rate of N fertilizer not only leads to waste of resources and environmental pollution, but also seriously threatens human health. Therefore, it is very important to improve plant N use efficiency (NUE) and to reduce environmental pollution. Transport from soil to root and from root to shoot and other plant organs involves many processes such as N uptake, assimilation, transport, and reuse. N assimilation is not only the most critical step in these processes, but also one of the most important limiting factors for plant growth. Therefore, improving N assimilation efficiency is an important aspect of improving plants’ NUE.

In several plant species, a part of the nitrate absorbed by the roots is assimilated in the roots, but most of it is transported to the shoot and then assimilated. Nitrate is first reduced to nitrite by nitrate reductase (NR) in the cell cytoplasm, and previous studies have shown that the activity of NR is regulated by 14–3-3 proteins, protein kinases, proteases, and protein phosphatases [21]. This enzyme is regulated by NR [NADH] proteins (NIAs), and NIA2, rather than NIA1, regulates the NR activity in *A. thaliana* [18, 22]. The reduction of nitrite to ammonium by nitrite reductase requires NAD(P)H. In addition, the assimilation of ammonium to amino acids via the glutamine synthetase (GS)/NADH-glutamine oxoglutarate transaminase (NADH-GOGAT) cycle also requires ATP and NADH or reduced ferredoxin [18]. Recent studies have shown that NIN-like protein transcription factors are key regulators of nitrate-induced *NR* gene expression and that NIN-like protein transcription factors may be in harmony with nitrate-induced expression of other nitrate assimilation-related genes [23, 24].

The ammonium restored from nitrate or absorbed directly by the action of ammonium transporters (AMTs) is further reduced by nitrite reductase in the plastid and by GS in the plastid and cytoplasm [25] or assimilated into amino acids through the GS and GOGAT cycles. The main GS/GOGAT isozymes involved in these processes are GS2 and ferredoxin-dependent GOGAT (Fe-GOGAT) in the chloroplast, and GS1 and NADH-GOGAT in the cytoplasm [26, 27]. The physiological functions of some GS1 isoenzymes in *A. thaliana* have been reported [28–30]. In this species, GLN1.1 and GLN1.4 showed high affinity for ammonium, while GLN1.2 and GLN1.3 showed low affinity [31]. At low concentrations, ammonium is assimilated by GLN1.1, GLN1.2, and GLN1.3, and they are functionally redundant [32]. Some studies in maize also pointed out that GLN1.4 could work on the re-assimilation of the released ammonium [33, 34]. In *A. thaliana*, GLN1.5 has not been detected at the transcription level [35] whereas GLN2-encoded GS has double targeting to leaf mitochondria and chloroplasts [36]. In addition to the major N assimilation, N re-assimilation also captures large amounts of ammonia through photorespiration in photosynthetic tissues and protein conversion during senescence or seed germination [37].

Glutamate metabolism is related to glutamate dehydrogenase (GDH) activity, which catalyzes the interconversion of glutamate and 2-oxoglutarate. Recent studies in *A. thaliana* clearly showed that GDH plays a central role in amino acid decomposition under carbon deficiency, and the main physiological function of NADH-GDH is to provide 2-oxoglutarate for the tricarboxylic acid cycle [38, 39].

N assimilation is a reaction to internal and external clues of N metabolites such as amino acids, ammonium, and nitrates. Isozymes are regulated at the level of transcription, translation, and post-translational modification [40]. The regulation of N uptake and assimilation is also related to root development. Absorption is particularly dependent on root-related characteristics, because plants not only regulate their metabolism and gene expression through their roots, but also adjust their structure to optimize resource acquisition [41, 42].

In a changing environment, a more comprehensive understanding of N assimilation and its regulation is of great importance to improve plant productivity. Therefore, it is necessary to strengthen basic research on reference species as well as on other plant species. In the present study, and by examining the molecular and genetic mechanism of N assimilation in *A. thaliana*, we explored the role of *AtPDF2.1* on ammonium metabolism regulation.

## Results

### *AtPDF2.1* response to ammonium

**Fig. 1:**
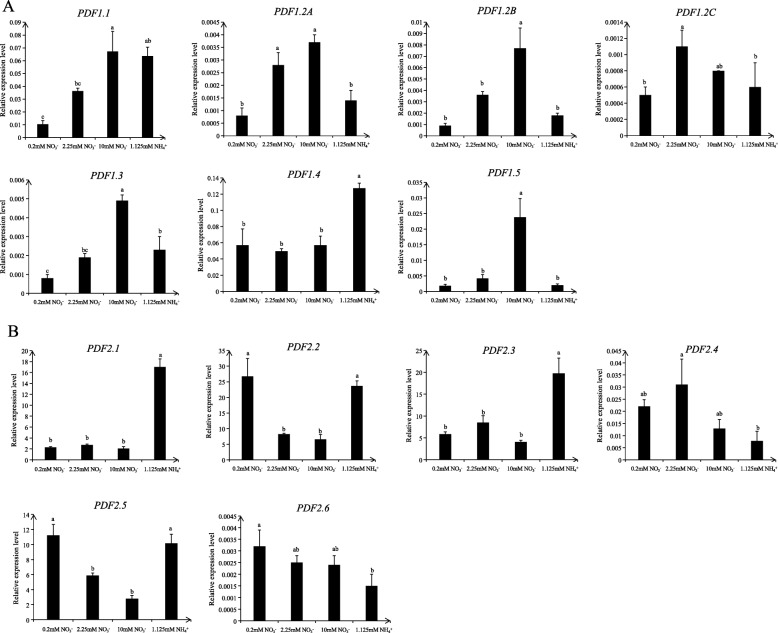
Analysis of the relative expression level of *AtPDF* family genes in the roots of *A. thaliana* under different treatments. The seedlings were treated with 1/4 plant nutrient solution for 18 d, N-starved for 3 d, and then treated with 0.2 mM KNO_3_, 2.25 mM KNO_3_, 10 mM KNO_3_, or 1.125 mM NH_4_NO_3_ for 6 h before roots were harvested separately for RNA analysis. **a** *AtPDF1*s. **b** *AtPDF2*s. *Actin2* was used as the internal control in the quantitative real-time PCR. Bars displaying the same letters are not significantly different at *P* < 0.05 according to the least significant difference test

**Fig. 2:**
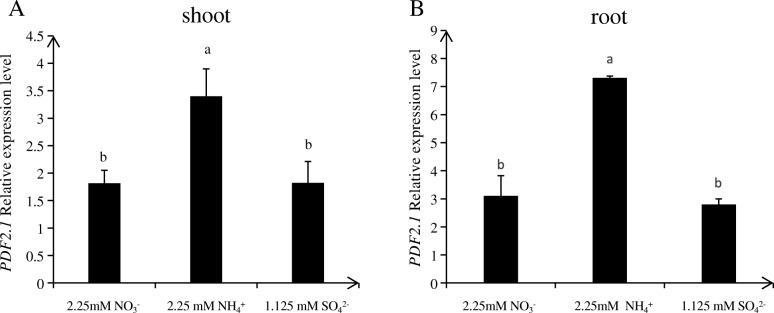
*AtPDF2.1* relative expression is significantly induced by ammonium. *A. thaliana* seedlings were treated with 1/4 plant nutrient solution for 18 d and then N-starved for 3 d. The seedlings were treated with 2.25 mM KNO_3_, 1.125 mM (NH_4_)_2_SO_4_, and 1.125 mM K_2_SO_4_ for 6 h before roots and shoots were harvested separately for RNA analysis. **a** shoot. **b** root. *Actin2* was used as an internal control. Bars displaying the same letter are not significantly different at *P <* 0.05 according to the least significant difference test

First, we conducted induction experiments on all members of the *PDF* family genes to assess their responses to high nitrate, low nitrate, and ammonium. The results obtained for Col-0 hydroponically grown for 18 d under culture conditions, N-starved for 3 d, and then treated with 0.2 mM KNO_3_, 2.25 mM KNO_3_, 10 mM KNO_3_, or 1.125 mM NH_4_NO_3_ for 6 h before root sampling showed that *PDF2.1* and *PDF2.3* were induced by ammonium, particularly *PDF2.1* (Fig. 1). To further verify if *PDF2.1* was induced under pure ammonium growth conditions, we used the wild type Col-0 hydroponically grown for 18 d under culture conditions, N-starved for 3 d, and then treated with 2.25 mM KNO_3_, 1.125 mM(NH_4_)_2_SO_4_, or 1.125 mM K_2_SO_4_ for 6 h before shoot and root sampling. This experiment revealed that *AtPDF2.1* was significantly induced under the ammonium treatment both in shoot and root (Fig. 2).

### *AtPDF2.1* is mainly expressed in the root vascular bundles and cotyledons and its protein is localized to the cell wall

**Fig. 3:**
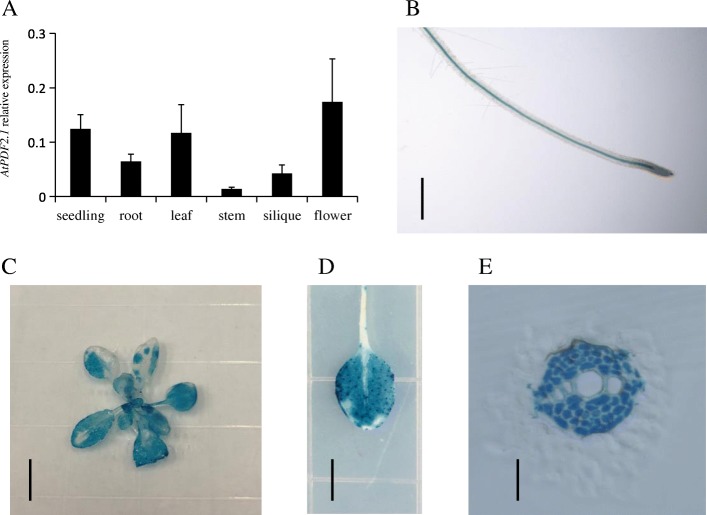
Expression patterns of *AtPDF2.1*. *AtPDF2.1* is mainly expressed in shoot and root vascular bundles. **a** Analysis of the relative expression level of AtPDF2.1 in different organs of *A. thaliana* by quantitative real-time PCR. Tissues were harvested either from 45-d-old plants grown in hydroponics (leaves, stems, siliques, and flowers) or from 7-d-old plants grown in 1/2 MS medium (seedlings). *Actin2* was used as an internal control. Data are mean ± SD, *n* = 4. **b**–**e** Histochemical localization of GUS activity in transgenic plants expressing the *GUS* reporter gene under the control of the pro*AtPDF2.1* promoter. **b** One-week-old whole-mount seedling root. **c**, **d** 45-d-old plants shoot (**c**) and leaves (**d**). **e** Cross-section of one-week-old root. Bars = 4 mm in (**b**), 2 cm in (**c**), 1 cm in (**d**), and 10 μm in (**e**)

**Fig. 4:**
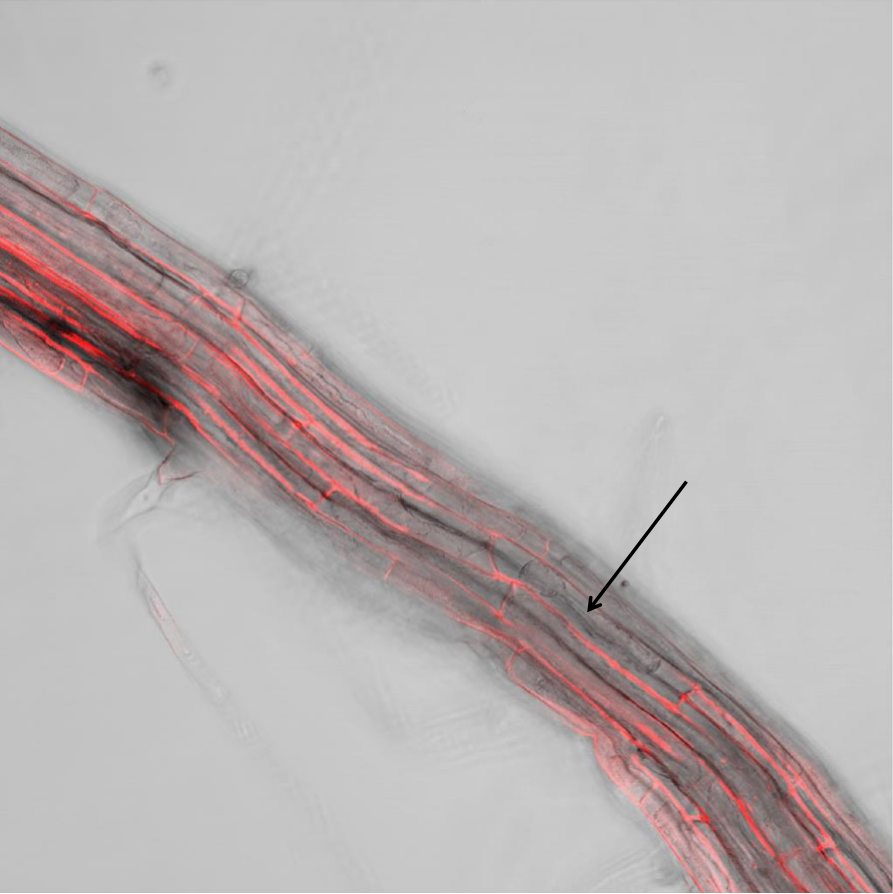
Confocal observation of red fluorescence in 35S::*AtPDF2.1-mRFP* transgenic *A. thaliana*. Subcellular localization merge map. Surface sterile seeds were plated onto 1/2 Murashige and Skoog medium and grown vertically for 2 weeks. The root epidermal cells were incubated in 40% sucrose to induce plasmolysis, and then imaged by confocal microscopy. The arrow points to the plasmic wall separation

To elucidate the expression pattern of *AtPDF2.1*, we generated *AtPDF2.1* promoter-driven β-glucuronidase (GUS) transgenic plants. *PDF2.1* was expressed in the root, seedling, leaf, stem, silique, and flower, as determined by quantitative PCR analysis (Fig. 3a). We detected strong GUS signals in the leaves, cotyledons, and root vascular bundles (Fig. 3b-d). The cross-section GUS analysis showed strong expression in parenchyma cells of vascular xylem in the roots (Fig. 3e). To determine the subcellular localization of *AtPDF2.1*, we transformed *A. thaliana* plants with *AtPDF2.1-mRFP* using the 35S promoter. The subcellular localization assays showed that the fluorescence signal was predominant in the cell wall of transformed plants (Fig. 4). These results indicated that AtPDF2.1 is localized to the cell wall.

### *AtPDF2.1* affects the concentration of ammonium in shoots

**Fig. 5:**
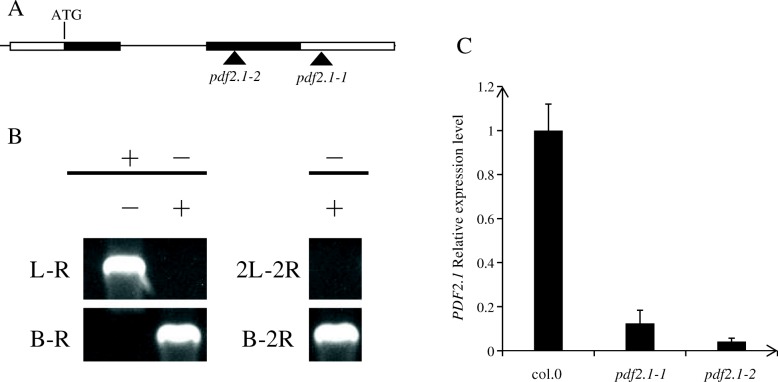
Identification of *pdf2.1* mutants. **a** Schematic representation of the allelic transfer DNA insertion lines *pdf2.1–1* (SALK_110286) and *pdf2.1–2* (SALK_206700C).The white blocks represent 5′-3′ UTR and the line connected the two black blocks represents intron. **b** Identification of homozygote mutants by PCR screening and sequencing. The first column is wild type, and the second and third columns are screened homozygotes. L and 2 L represent the left primer of two mutants, R and 2R represent the right primer of two mutants, and B represents the inserted sequence. **c** Quantitative real-time PCR failed to detect intact AtPDF2.1 mRNA in *pdf2.1* mutant plants. *Actin2* was used as an internal control

**Fig. 6:**
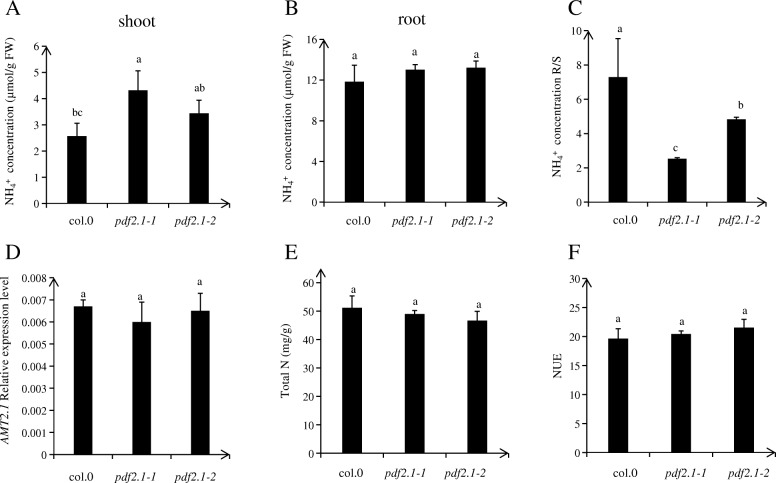
Functional disruption of *AtPDF2.1* increases ammonium accumulation in shoot. **a**, **b** Ammonium concentration in Col-0, *pdf2.1–1*, and *pdf2.1–2* plants grown in 1/4 plant nutrient solution for 21 d in (**a**) shoot and (**b**) root. **c** Ammonium concentration ratio of root to shoot. **d** *AMT2.1* gene expression in roots of Col-0, *pdf2.1–1*, and *pdf2.1–2.* **e** Total N content of Col-0, *pdf2.1–1*, and *pdf2.1–2* plants grown in 1/4 Plant Nutrient Solution for 21 d. Total N amount was obtained from pooled samples of six plants from each replicate. **f** Nitrogen use efficiency (NUE), NUE = total dry weight/total N content. *Actin2* was used as an internal control. Data represent means ± SEs (*n* = 4). Bars displaying the same letters are not significantly different at *P <* 0.05 according to the least significant difference test

We first screened and identified the *AtPDF2.1* mutants. Although the expression level of *AtPDF2.1* in the mutants was significantly lower than that in the wild type, the expression of this gene was still detected. Therefore, we obtained two functional knock down mutants, *pdf2.1–1* and *pdf2.1–2* (Fig. 5). After transplantation, Col-0, *pdf2.1–1*, and *pdf2.1–2* seedlings were cultured under normal conditions (1/4 plant nutrient solution) for 18 d and then sampled and analyzed for differences in total N content, NUE, ammonium concentration, and gene expression of *AMT2.1* between the selected *AtPDF2.1* mutants and Col-0. Under normal conditions, ammonium concentrations were higher in *pdf2.1–1* and *pdf2.1–2* shoots than in Col-0 shoots, but no significant differences were detected among roots (Fig. 6a, b). There were also no significant differences in nitrate concentrations in the shoots and roots of Col-0, *pdf2.1–1*, and *pdf2.1–2* plants (Additional file 1: Figure S1). Protein AMT2.1 transports ammonium from the root to the shoot [43, 44]. Under normal conditions, there was no difference in *AtAMT2.1* expression in the roots of Col-0, *pdf2.1–1*, and *pdf2.1–2* (Fig. 6d), indicating that *AtPDF2.1* was not involved in ammonium transport from roots to shoots. The total N content and NUE of Col-0, *pdf2.1–1*, and *pdf2.1–2* plants also showed no differences (Fig. 6e, f). These results suggested that *AtPDF2.1* may affect the metabolism of ammonium in shoots.

### *AtPDF2.1* affects GS activity by regulating the expression of *GLN1.3* and *GLN1.5* in shoots

Although *AtPDF2.1* had no effect on total N content, root ammonium concentration, and expression of *AtAMT2.1*, ammonium concentration was significantly higher in the shoots of mutant *A. thaliana* than in Col-0 shoots. Therefore, we hypothesized that *AtPDF2.1* might participate in the regulation of ammonium metabolism in the shoot.

**Fig. 7:**
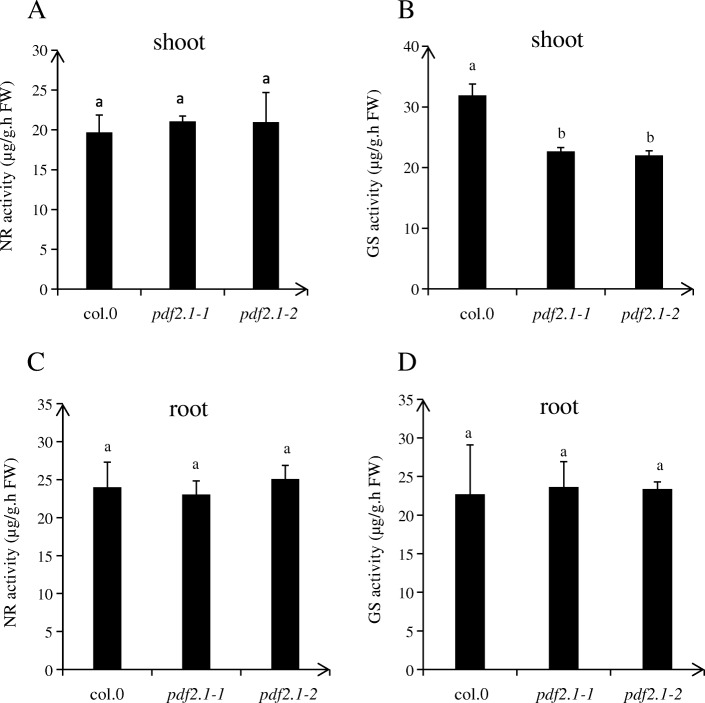
Functional disruption of *AtPDF2.1* decrease GS activity in shoot. Plants were grown in 1/4 plant nutrient solution for 21 d. **a** NR and (**b**) GS activities in the shoots of Col-0, *pdf2.1–1*, and *pdf2.1–2*, **c** NR and (**d**) GS activities in the roots of Col-0, *pdf2.1–1*, and *pdf2.1–2*. Data represent means ± SEs (*n* = 4). Bars displaying the same letters are not significantly different at *P <* 0.05 according to the least significant difference test

**Fig. 8:**
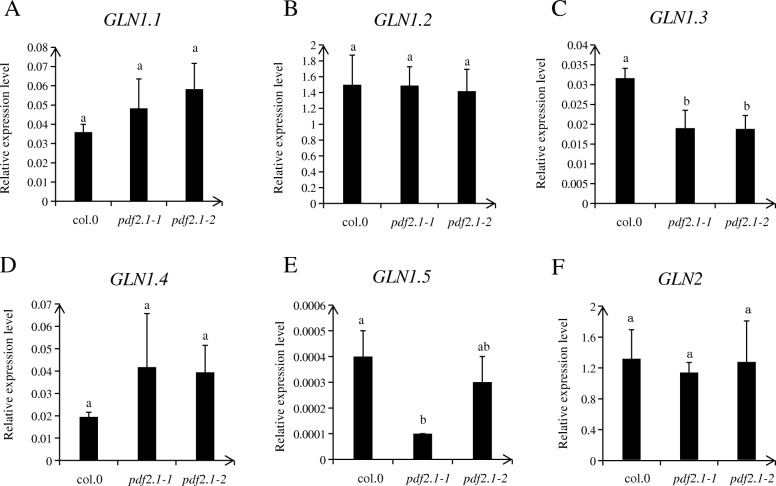
*GLN1.3* and *GLN1.5* are down regulated in shoots of *pdf2.1* mutant. Plants grown in 1/4 plant nutrient solution for 21 d. Shoots were sampled to examine the expression levels of (**a**) *GLN1.1*, **b** *GLN1.2*, **c** *GLN1.3*, **d** *GLN1.4*, **e** *GLN1.5*, and **f** *GLN2*. Data represent means ± SEs (*n* = 4). *Actin2* was used as an internal control. Bars displaying the same letters are not significantly different at *P <* 0.05 according to the least significant difference test

Under normal conditions, there was no difference in NR activity among the shoots of Col-0, *pdf2.1–1*, and *pdf2.1–2*, while GS activities in the shoots of *pdf2.1–1* and *pdf2.1–2* were significantly lower than in the shoot of Col-0. In addition, ammonium concentration in the shoots of *pdf2.1–1* and *pdf2.1–2* was higher than in Col-0 shoots (Fig. 7). In *pdf2.1* mutants, *GLN1.3* and *GLN1.5* were downregulated, but no effect was detected for other *GLN* family genes. Thus, *AtPDF2.1* may affect the assimilation of ammonium into glutamine by regulating the expression of *GLN1.3* and *GLN1.5* (Fig. 8).

### *AtPDF2.1* affects ammonium metabolism in shoots

**Fig. 9:**
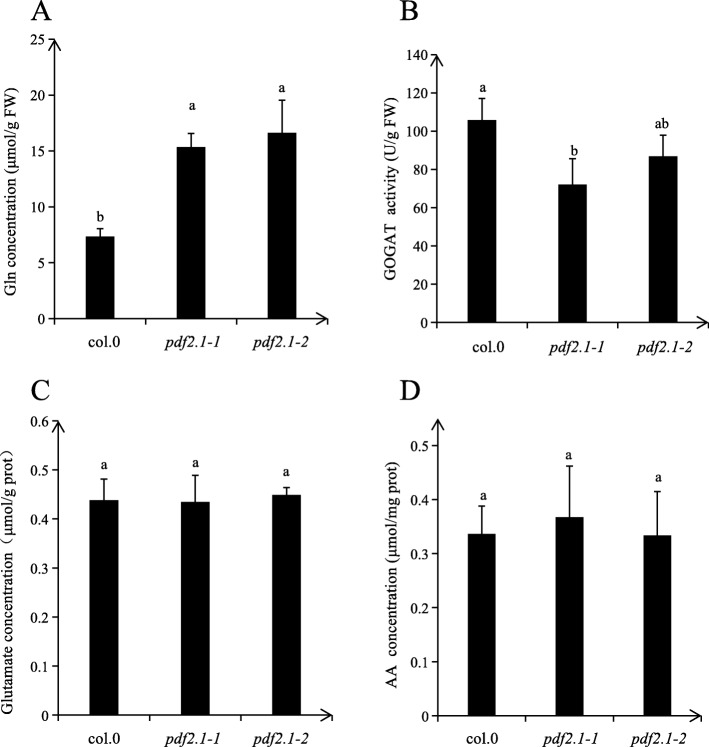
Activities of ammonium assimilation enzymes and concentration of ammonium assimilation products in shoots of Col-0 and *pdf2.1* mutants. Plants were grown in 1/4 plant nutrient solution for 21 d. **a** Concentration of glutamine (Gln). **b** Activity of glutamate synthase (NADH-GOGAT). **c** Concentration of glutamate (Glu). **d** Concentration of total amino acids (AA) in shoots of Col-0, *pdf2.1–1*, and *pdf2.1–2*. Data represent means ± SEs (*n* = 4). Bars displaying the same letters are not significantly different at *P <* 0.05 according to the least significant difference test

To examine which steps of ammonium metabolism are specifically affected by *AtPDF2.1*, we determined the concentrations of glutamine and glutamic acid, the activity of NADH-GOGAT, and the concentration of free amino acids. Glutamine concentrations in *pdf2.1–1* and *pdf2.1–2* were lower than in Col-0, and NADH-GOGAT activity was also lower in the mutants than in Col-0 (Fig. 9a, b). Thus, the decreased GS activity in mutants might have affected subsequent metabolic pathways. There were no significant differences in the concentrations of glutamic acid and free amino acids among Col-0, *pdf2.1–1*, and *pdf2.1–2* (Fig. 9c, d), possibly due to the functional redundancy of *GLN1.3* and *GLN1.1* and extremely low expression of *GLN1.5*, leading to a dominant role of *GLN1.2* [32, 34].

Overall, results suggest that *AtPDF2.1* regulates the activity of GS by altering the expression of *GLN1.3* and *GLN1.5*, leading to changes in ammonium assimilation in shoots. This alters glutamine concentration and NADH-GOGAT activity, thereby affecting the assimilation of ammonium.

## Discussion

PDFs are small cysteine-rich peptides firstly found in wheat and barley seeds [45]. In *A. thaliana*, defensins are divided into two families, PDF1s and PDF2s. A previous study showed that AtPDF1.1 is involved in plants’ response to biotic stress [46]. Other studies found that PDF1s increased zinc tolerance of plants and yeasts [9, 47, 48]. However, to our knowledge, the molecular mechanism of PDFs had not been clarified.

Some reports found that small peptides are associated with N. In *A thaliana* under nitrate deficiency, the expression of *CEP3* in roots increased by 10-fold and *CEP1* expression in seedlings also increased; under ammonium restriction, *CEP9* expression was inhibited [15]. Recent studies revealed that CEP family peptides are part of the systemic N-demand signal transduction in roots. They sense signals through their receptor CEPRs, thereby mediating the systemic up-regulation of nitrate transporter genes in roots [13]. However, whether PDFs play a regulatory role in nutrient uptake, transport, or assimilation has not been reported. In the present study, the wild type Col-0 was treated with low nitrate, high nitrate, and ammonium, and the expression of *AtPDF* family genes under these conditions was analyzed. We found that most *PDF1*s responded to nitrate, while *PDF2.1* and *PDF2.3* were induced by ammonium, especially *PDF2.1* (Fig. 1). It has been reported that PDF2.1 and PDF2.3 have high homology [2], and we found that PDF2.1 and PDF2.3 are similarly induced by ammonium, suggesting they might have some functional redundancy, which would explain why phenotypic differences were not obvious between wildtype and PDF2.1 mutants. Recently, we obtained homozygous mutants of PDF2.3 through the clustered regularly interspaced short palindromic repeats (CRISPR)/CRISPR-associated protein 9 technology, and we aim to carry out a series of tests on *pdf2.3* single or *pdf2.1*/*pdf2.3* double mutants and combine those results with previous ones to gain insight on the functioning of PDF2.1 and PDF2.3. In the present study, *PDF2.1* was induced under pure ammonium culture conditions (Fig. 2). PDFs are expressed in the xylem, stomata and stomatal cells, parenchyma cells, and other peripheral regions [1, 49]. Our results also confirmed that AtPDF2.1 is a cell wall protein of *A. thaliana*, which is expressed in all tissues. However, because we did not know how *AtPDF2.1* regulates N and/or ammonium metabolism in *A. thaliana*, we examined Col-0 and *PDF2.1* mutant responses of *AtPDF2.1* under normal culture conditions.

Firstly, we determined the concentration of ammonium in Col-0, *pdf2.1–1*, and *pdf2.1–2*. We found no significant differences in roots between Col-0 and the mutants, but ammonium concentration was significantly higher in the shoots of mutants than in the shoots of Col-0. However, no significant differences were detected in the expression of *AtAMT2.1* between Col-0 and mutants (Fig. 6), indicating that *AtPDF2.1* did not affect ammonium transport from roots to shoots. However, no significant differences were observed in shoot and root nitrate concentrations between Col-0 and *pdf2.1* mutants (Additional file 1: Figure S1), and there were no differences in the total N content and NUE (Fig. 6). Therefore, *AtPDF2.1* might affect ammonium metabolism in shoots. The GS enzyme located in the chloroplast and cytoplasm is responsible for assimilating ammonium, which is produced via nitrate reduction [9, 47, 48].

We measured the activities of enzymes related to N metabolism in shoots and found that there were no significant differences in NR activities in shoots, while GS activities were significantly lower in *pdf2.1–1* and *pdf2.1–2* than in Col-0. This suggested that *AtPDF2.1* may regulate the further metabolism of ammonium by regulating GS activities. The *pdf2.1* mutants showed no effect of most *GLN* family genes but displayed regulatory effects for *GLN1.3* and *GLN1.5*. In addition, the relative expression of *GLN1.5* was low. This might explain why the phenotype was not obvious. A recent study indicated that *GLN1.1*, *GLN1.2*, *GLN1.3*, and *GLN1.4* are functional members of the *GLN1* gene family in *A. thaliana* and that they play a synergistic or complementary role in primary N assimilation, plant growth, seed germination and production, and pollen development [50]. In addition, the major isoforms of *A. thaliana* expressed in the seedling stage are GLN1.1, GLN1.2, and GLN1.3 [43]. Other studies have reported that GLN1.2 plays a significant role in seed yield, lotus rosette biomass, and germination [28, 30], and that the loss of GLN1.1 and GLN1.3 function reduced the germination rate of plants [50]. This might be why PDF2.1 mutants only partially affected ammonium metabolism.

In this experiment, the concentration of glutamine was higher in mutants than in the wild type, likely due to the decrease of NADH-GOGAT activity in the mutants. However, the expression of glutamate transporter 1 (*GLT1*), the gene regulating NADH-GOGAT, showed no difference between wild type and mutants (Additional file 1: Figure S2). Thus, this regulation might not occur at the transcriptional level. The decrease of GS activities affected the subsequent metabolic process. However, the glutamate and free amino acid concentrations did not differ significantly between Col-0 and mutants. This might be due to functional redundancy between *GLN1.3* and *GLN1.1* [50], low expression of *GLN1.5*, or *GLN1.2* playing a leading role among *GLN1.1*, *GLN1.2*, and *GLN1.3* [51].

Overall, PDF2.1, a cell wall protein, affects ammonium metabolism by regulating the nuclear gene *GLN1.3*. Several studies revealed that small peptides can be used as signal molecules of protein kinase pathways to indirectly regulate the expression of other genes [13, 14, 52]. For example, the small peptide CLE25 can regulate the expression of NCED3 in the leaves through the receptor kinase BAM, and thus transmits the signal of water shortage, affecting abscisic acid biosynthesis and transpiration and regulating stomata [14]. It has also been suggested that the CEP family peptides are a part of N-signaling in the root system. They sense signals through two LRR markers, namely CEPR1 and CEPR2, to adjust the systemic upregulation of nitrate transporter genes in the roots [13]. It has been shown that root-derived CEP induces the phloem-specific polypeptides CEPD1 and CEPD2 in the leaves and activates NRT2.1 expression, especially during nitrate uptake by the roots [52]. Therefore, we hypothesize that PDF2.1 can also regulate GLN1.3 and AMT2.1 through a protein kinase or downstream transcription factor, thus affecting ammonium metabolism, but the specific process has not been determined.

Based on these results, we believe that *AtPDF2.1* regulates the metabolism of ammonium by regulating the activities of GS in the shoot, thereby affecting the concentration of glutamine and the activity of NADH-GOGAT.

## Conclusions

Our results showed that PDF2.1 is a cell wall protein in various organs, and that it affects the metabolism of ammonium by regulating the expression of *GLN1.3* in plant shoots.

## Methods

### Experimental materials and growth conditions

The *A. thaliana* wild type (Col-0) used as the control group in all experiments was provided by Jiming Gong from Shanghai Institute of Plant Physiology and Ecology. The *AtPDF2.1* knockout mutants (*pdf2.1–1* and *pdf2.1–2*) were obtained from The Arabidopsis Information Resource (TAIR; http://www.arabidopsis.org/). The seeds of Col-0 and *AtPDF2.1* knockout mutants were germinated and grown in a greenhouse (300 μmol photons m^− 2^ s^− 1^, 16 h photoperiod, 22 °C) for 10 d. A pair of real leaf seedlings was transplanted into a 4.5-L pot and cultured for 21 days in 1/4 plant nutrient solution containing 1.25 mM KNO_3_, 0.625 mM KH_2_PO_4_, 1.25 μM Fe-EDTA, 0.5 mM MgSO_4_, 0.5 mM Ca(NO_3_)_2_, 0.05 μM NaMoO_4_, 0.125 μM CuSO_4_ 0.25 μM ZnSO_4_, 3.5 μM MnCl_2_, and 17.5 μM H_3_BO_3_. The pH of the medium was adjusted to 5.8, and MES (2.5 mm) was added to the buffer to adjust any possible change in pH. The medium was renewed every 4 d. Forty-eight plants were planted in each basin, and the growth conditions of all basins were the same.

In the ammonium induction experiments, set to analyze the relative expression levels of *AtPDF2.1* under different treatments, Col-0 seedlings were treated with 1/4 plant nutrient solution for 18 d and then subject to N starvation for 3 d. After this period, seedlings were treated with 2.25 mM KNO_3_, 1.125 mM (NH_4_)_2_SO_4_, or 1.125 mM K_2_SO_4_ for 6 h, before roots and shoots were harvested separately for RNA extraction and *AtPDF2.1* expression analysis.

In other experiments, Col-0, *pdf2.1–1*, and *pdf2.1–2* seedlings were treated with 1/4 plant nutrient solution for 21 d and then sampled and analyzed.

### Histochemical analysis

The 1805-bp genomic fragment immediately upstream of the initial codon of AtPDF2.1 was amplified by PCR using primer ProAtPDF2.1 (Additional file 1: Table S1). Then, subcloning the generated ProAtPDF2.1 promoter split into binary vector pCAMBIA1300 [53]. The *A. thaliana* seedlings cultured in water for 21 d were sampled as reported in section “Experimental materials and growth conditions”. Semi-thin sections (4 μm) were cut from the root, fixed on slides, and observed under the Leica-DM6000 microscope. Histochemical staining driven by the ProAtPDF2.1 promoter was performed using the GUS histochemical analysis kit (Real-Times). The staining pattern of GUS in the root was observed under the Olympus BX51 microscope and photographed using the Fujifilm X-A3 camera.

### DNA constructs and transformation into plants

The coding sequence of *AtPDF2.1* was amplified by PCR using primers AtPDF2.1F and AtPDF2.1R (Additional file 1: Table S1), and then the subcellular location of AtPDF2.1 in *Arabidopsis* was determined. Subcloning was performed to generate construct 35S::mRFP/1300. The resulting fragment was framed with the 5 ‘end of the single red fluorescent protein (mRFP) gene to produce the 35S::*AtPDF2.1-mRFP*/pCAMBIA1300 constructs. These constructs were modified by replacing the 35S promoter with the native promoter proAtPDF2.1, resulting in the proAtPDF2.1::*AtPDF2.1-mRFP*/pCAMBIA1300 constructs, which were transformed into *A. thaliana* using the floral dip method [54]. The root tissue of transgenic plants was then imaged by mRFP using a confocal microscope (LSM880; Zeiss).

### Quantification of N concentrations

Using hydroponics, after 21 d, the shoots and roots of Col-0 and mutant *A. thaliana* plants were individually sampled, frozen in liquid N, and stored at − 80 °C until further analysis. Indophenol blue colorimetry, at 630 nm [55–57] and using (NH_4_)_2_SO_4,_ was performed to measure ammonium concentration. Nitrate concentration in the roots and leaves was determined at 410 nm [57–59] spectrophotometrically. Total N concentration was determined as described by Wang et al. [60]. In the present study, NUE was determined as total biomass/total N accumulation [61].

### Nitrogen and ammonium metabolism-related enzyme activities

N metabolism in plants is closely associated with the activities of several key enzymes, such as NR and GS [62]. For NR activity determination, the roots and leaves harvested were frozen in liquid N immediately, and then stored at − 80 °C until further analysis. Samples were ground to a fine powder (~ 100 mg), extracted, and analyzed spectrophotometrically [57, 63, 64]. The activity of GS was assayed as reported by Wang et al. [65]. The activity of NADH-GOGAT was quantified using a NADH-GOGAT measurement kit (Solarbio Bioengineering Institute). Glutamate and glutamine were quantified using a glutamic acid measurement kit and a glutamine measurement kit, respectively (both from Nanjing Jiancheng Bioengineering Institute). Enzyme activities were expressed as moles of metabolite generated/consumed per milligram of fresh weight or protein per unit of time. The protein concentration was determined by the Coomassie brilliant blue method with Modified BCA Protein Assay Kit, C503051, Sangon Biotech.

### Amino acids quantification

High-performance liquid chromatography (HPLC) was used to quantify amino acids in the shoots as reported by Del Campo et al. [66]. Frozen leaf samples (200 mg) were pulverized with liquid N and homogenized in 1.5 mL of 0.1% phenol and 6 M HCl. The homogenate was hydrolyzed for 22 h at 100 °C, and then cooled. One milliliter of the hydrolysate was dried using NDK200–2 organomation (Hangzhou MIU Instrument Co., Ltd.) and re-dissolved in 1 mL of 0.1 M HCl. To quantify the amino acids, 200 μL of the re-dissolved hydrolysate was mixed with 20 μL of norleucine internal standard solution, 200 μL of triethylamine acetonitrile (pH > 7), and 100 μL of isothiocyanate acetonitrile, and the mixture was incubated at 25 °C for 1 h. After adding 400 μL of hexane, the sample was incubated for another 10 min with shaking. The solution in the underlay was passed through a 0.45-μm syringe filter. All HPLC analyses were performed on the RIGOL L3000 system (Beijing RIGOL Technology Co., Ltd.). Chromatographic separation was accomplished using an RP-HPLC ACE column (5C18-HL) with a particle size of 5 μm (250 mm × 4.6 mm), at 100 °C through a binary gradient. Mobile phase A was 25 mM acetate buffer (pH 6.5) and 70 mL acetonitrile. Mobile phase B was 80% acetonitrile aqueous solution. The flow rate was 1.0 mL min^− 1^ and the column temperature was 40 °C.

### Genotyping, RNA extraction, and quantitative PCR

To identify the mutants, the total DNA was extracted from 21-d-old plants grown in 1/2 plant nutrient solution (leaves), which were used as templates in the PCR with the primers presented in Additional file 1: Table S1.

In the ammonium induction experiments, the roots and shoots of Col-0 seedlings, which were treated with 1/4 plant nutrient solution for 18 d, and then N-starved for 3 d before treatment, were separately harvested, frozen in liquid N, and stored at − 80 °C until RNA analysis. To analyze the expression pattern of *PDF2.1*, tissues were harvested from 45-d-old plants grown in hydroponics (leaves, stems, siliques, and flowers) or from 7-d-old plants grown in 1/2 plant nutrient solution (seedlings). The total RNA was extracted with TRIzol (Invitrogen), precipitated with an equal volume of isopropanol, washed with 75% ethanol, and dissolved in RNase-free water, according to the manufacturer’s instructions. Complementary DNA was synthesized using the PrimeScript™ RT Kit with gDNA Eraser (Perfect Real Time; TAKARA) following the protocol of the manufacturer. The relative expression of the target genes was determined by quantitative real-time PCR performed on an Applied Biosystems StepOne™ Real-Time PCR System with SYBR Premix Ex-Taq (TAKARA), according to the manufacturer’s instructions. The relative expression of the target genes was normalized to that of the reference gene using the 2^-ΔΔCT^ method [67]. Primers used in the assays are listed in Additional file 1: Table S1, and the expression data were normalized to that of *Actin2*, which was used as the internal standard.

### Statistical analyses

In this study, all experiments were in progress using a completely randomized design. Four biological replicates and two technical replicates were applied for each treatment. The least significant difference multiple range test was used to perform multiple comparisons. Differences between the wild type and mutants were evaluated using Student’s *t*-test with Statistical Productions and Service Solutions 17.0 (SPSS, Chicago, IL, USA). These Differences were considered statistically significant at *P* < 0.05.

## Supplementary information

**Additional file 1:** **Figure S1.** Nitrate concentration in *A. thaliana*, **Figure S2.** Relative expression level of *AtGLT1* in the shoots of of Col-0, *pdf2.1–1*, and *pdf2.1–2 A. thaliana* plants grown in 1/4 plant nutrient solution for 21 d, **Table S1.** Primers used in the present study

## Data Availability

The datasets used and/or analyzed during the current study are available from the corresponding author on reasonable request.

## Abbreviations

AMT: Ammonium transporters
GDH: Glutamate dehydrogenase
GOGAT: Glutamine oxoglutarate transaminase
GS: Glutamine synthetase
N: Nitrogen
NR: Nitrate reductase
NUE: Nitrogen use efficiency
PDF: Plant defensins
