# Supplementary material for: The plant defensin gene AtPDF2.1 mediates ammonium metabolism by regulating glutamine synthetase activity in Arabidopsis thaliana
Source: BMC Plant Biol. 2019 Dec 16;19:557. doi: 10.1186/s12870-019-2183-2 (PMC6916093; doi:10.1186/s12870-019-2183-2)
Supplement: Supplementary file 1 — Additional file 1: Figure S1. Nitrate concentration in A. thaliana, Figure S2. Relative expression level of AtGLT1 in the shoots of of Col-0, pdf2.1–1, and pdf2.1–2 A. thaliana plants grown in 1/4 plant nutrient solution for 21 d, Table S1. Primers used in the present study [file 12870_2019_2183_MOESM1_ESM.docx]

Supplementary Material

**Supplementary Figure 1.** Nitrate concentration in *Arabidopsis thaliana*. (**A,B**) Nitrate concentration of Col-0, *pdf2.1-1*, and *pdf2.1-2* plants grown in 1/4 plant nutrient solution for 21 d in (A) shoot and (B) root. Data represent means ± standard errors (n = 4). Bars displaying the same letters are not significantly different at P < 0.05 according to the LSD test.

**Supplementary Figure 2.** Relative expression level of *AtGLT1* in the shoots of of Col-0, *pdf2.1-1*, and *pdf2.1-2* *Arabidopsis thaliana* plants grown in 1/4 plant nutrient solution for 21 d. Data represent means ± standard errors (*n* = 4). *Actin2* was used as the internal control in the quantitative real-time PCR. Bars displaying the same letters are not significantly different at *P <* 0.05 according to the LSD test.

**Supplementary Table 1**. Primers used in the present study.

| Primer  Name | Forward  Sequence | | Reverse  Sequence |
| --- | --- | --- | --- |
| *GUS* | | | |
| ProAtPDF2.1 | CGACGGCCAGTGCCAAGCTTA  TGCCTTATTGATACTGCGG | GACTGACCACCCGGGGATCCG  AGAGAGACAGAGTTGGAAA | |
| *Subcellular localization* | | | |
| AtPDF2.1 | CGGGGGACTCTAGAGGATCCA  TGAAGTTCTCTATGCGTTT | | TCGGAGGAGGCCATACTAGTG  CAATTTCTGGTGCAGAAGC |
| *Identification of mutants* | | | |
| LBb1.3 | ATTTTGCCGATTTCGGAAC | |  |
| SALK_110286 | LP：CTGGCGTTTCCATTTTTGTAG | | RP：GCAACTAGTCGTCTCCATTGC |
| SALK_206700C | LP：TTGTGGACTCCCCAATATCTG | | RP：AGATCGACGATTGAGTCATGC |
| *Real-time qPCR* | | | |
| *ACTIN2* | TGTGCCAATCTACGAGGGTTT | | TTTCCCGCTCTGCTGTTGT |
| *PDF1.1* | CACAGAAGTTGTGCGAGAGG | | GCAAGATCCATGTCGTGCTT |
| *PDF1.2A* | TCACCCTTATCTTCGCTGCT | | TTCTGTGCTTCCACCATTGC |
| *PDF1.2B* | ACCAACAATGGTGGAAGCAC | | ACTTGTGAGCTGGGAAGACA |
| *PDF1.2C* | ACCAACAATGGTGGAAGCAC | | TGCTCCCTCAAGGTTAATGC |
| *PDF1.3* | CCTCTTCGCTGCTCTTGTTC | | ACTTGTGAGCTGGGAAGACA |
| *PDF1.4* | ATGGCGGTGGAAGGAAGAAT | | GTGAAGCACGTTCCCATCTC |
| *PDF1.5* | TGAAGCACCGACAATTGTGAA | | CCACCAGCGCAATATCCATC |
| *PDF2.1* | ATATTCGTCGCCACAGGGAT | | CAGTTTGTATCGCTCACGCA |
| *PDF2.2* | GCGTGAGTGCATCAAACTG | | CAGCAATGTCTGGTGCAGAA |
| *PDF2.3* | GGTTCAAGGGTCCATGTGTG | | TGTCTTGTGCAGTAGCAACG |
| *PDF2.4* | ACAGTGATGGGTCTGGTCAC | | AAGGTCTGGTGCACAAACAG |
| *PDF2.5* | TCTCATCTCAGGAGATCATTGGG | | TCCCTCGTTACGGCAAACTA |
| *PDF2.6* | ATGTCGGTCATGCTCCTCTT | | TTTGGCACAGCTCTGTGAGT |
| *GLN1.1* | CAATGAGGGAAGAAGGCGGT | | CGCAACACCCCAAAGGAAAG |
| *GLN1.2* | CTTTCCTTTGGGGTGTTGCG | | AGCTGGCCTCCTATCCTCAA |
| *GLN1.3* | CACTGGAAAGCACGAAACCG | | CTGTGTCACGTCCCACTCTC |
| *GLN1.4* | AGATTGGAATGGTGCAGGGG | | TGTCACGCCCAACCCTAATC |
| *GLN1.5* | ATGGTGAAGGCAACGAGAGG | | GCCACTCCCCAAGAGAAAGT |
| *GLN2* | GATCGCCGTCCAGCATCTAA | | GGGCTTCAGCCTCAAGAGTT |
| *AMT2.1* | AGAAGATGAGATGGCAGGAATG | | AAACAGTCAAGGTCGGTGTAG |
